# Innovations in Phenotyping and Diagnostics Create Opportunities for Improved Treatment and Genetic Counseling for Rare Diseases

**DOI:** 10.3390/genes15060715

**Published:** 2024-05-31

**Authors:** Miles D. Thompson

**Affiliations:** Krembil Brain Institute, Toronto Western Hospital, 399 Bathurst Street, Toronto, ON M5T 2S8, Canada; miles.thompson@utoronto.ca

Genetic counseling and treatment options for rare developmental disabilities (DDs) have been revolutionized by the opportunities made possible by using massively parallel sequencing for diagnostic purposes. From the perspective offered by Thompson and Knaus [1] on the progress made in the diagnosis of glycophosphatidylinositol (GPI) biosynthesis defects (GPIBDs), such as Mabry syndrome, it is clear that the innovations in diagnostics may anticipate new treatment options emerging for some genetic DDs. In this context, Mausante et al. [2] highlight the fact that, in 2024, more than 50% of patients with epileptic encephalopathies (EEs) and developmental and epileptic encephalopathies (DEEs) will remain undiagnosed, largely due to their high degree of genetic heterogeneity and phenotypic variability. They discuss their dynamic diagnostic procedure, summarized in Figure 1, for investigating patients with previous negative or inconclusive genetic testing by whole-exome sequencing (WES), leading to a definite diagnosis in about 59% of participants.

This strategy, which can result in a high diagnostic yield, emphasizes the importance of the reverse phenotyping strategy and multidisciplinary teamwork to identify recessive and somatic variants, new genetic mechanisms, and complex genotype–phenotype associations [2]. The strategies could end invasive diagnostic procedures, facilitate precision medicine, provide comprehensive care for patients, and augment genetic counseling for affected families. This Special Issue presents a collection of case studies in diagnostics [3,4,5,6,7], a report of workflow for undiagnosed DDs [2], a review [8] and examination of fragile-X-related behaviors [9], and a study of the mechanism of action of remimazolam in malignant hyperthermia [10]. These diverse articles are complemented by the article by Morganthau et al. [11], who examine the benefits to care that can result from exposing medical students to DD patients.

The five case studies presented [3,4,5,6,7] illustrate the challenges associated with molecular diagnosis of DDs with phenotypic and genetic heterogeneity. For example, Thompson et al. [3] report a case report of a child with features of hyperphosphatasia with neurologic deficit (HPMRS; MIM 239300) for whom variants of unknown significance were identified in two post-GPI attachment to protein genes, *PGAP2* and *PGAP3*, that underlie HPMRS 3 and 4. The pathogenicity of these variants was established using a rescue assay of PGAP2 and PGAP3 functions in deficient CHO cell lines. Flow cytometric analysis showed that, while PGAP3 function was unaffected, CD59 and CD55 expression on the PGAP2-deficient cell line was not restored by the *PGAP2* variant. The phenotype of this patient with Mabry syndrome, therefore, was reported to be predominantly HPMRS3, resulting from autosomal recessive inheritance of NM_001256240.2 PGAP2 c:284A>G, p.Tyr95Cys [2]. By contrast, Khan et al. [4] used in silico modeling to identify likely pathogenic biallelic variants in the *NDST1* and *METTL23* genes in intellectual disability (ID), and German et al. [5] report an in silico workup of a novel *FA2H* variant identified as a homozygote in a male patient with a history that included childhood-onset progressive cognitive impairment and multisystem neurodegeneration consistent with FAHN/SPG35 upon brain imaging [5]. Case reports by Riviello et al. [6] and Callamare et al. [7] present evidence of chromosome structural changes that are associated with DD phenotypes. Riviello et al. report large genomic structural variation in DD phenotypes. An 8.5 Mb microdeletion at 2q37.1, extending to the telomere, and an 8.6 Mb interstitial microduplication at 2q34q36.1 were identified in a patient presenting with syndromic features. Their findings highlight 2q37 microdeletion syndrome, a subtelomeric deletion disorder characterized by variable-sized deletions associated with short stature, facial dysmorphism, and features of autism spectrum disorder [6]. Callamare et al. [7] report a balanced t(3;10) translocation and an 8.6 Mb 5q12 deletion, identified through array-CGH chromosome deletions, associated with a phenotype that includes postnatal growth retardation, intellectual disability, facial dysmorphism, and epilepsy. The *PDE4D* and *PIK3R1* genes were identified as the two major candidates responsible for the clinical features. The authors report that elements of the phenotype interpreted as “balanced” by conventional cytogenetics are mainly due to a cryptic deletion, highlighting the need for investigation prior to attributing cause to a cytogenetic rearrangement [7].

Two manuscripts examine aspects of fragile X syndrome (FXS). Fragile X syndrome (FXS), the most common single-gene disorder associated with ASD, results from LOF in the fragile X (FMR1) gene due to an unstable CGG repeat in the 5′ untranslated region (5′ UTR) [8,9]. 50% of males and 20% of females with LOF *FMR1* variants have features of autism spectrum disorder (ASD). Joga-Elvira et al. [9] analyze the relationship between executive functions and adaptive behavior in twenty-six girls with FXS and 14 controls in a school setting that was conducted at the Hospital Parc Tauli in Sabadell, Barcelona, Spain. The authors report that an alteration in the executive functions may affect the daily functioning of girls more than boys with FXS. Fyke et al. [8] report that among ASD patients, 3% test positive for FXS and discuss the role of FMRP, the protein encoded by *FMR1*, in ASD patients who do not have FXS.

The perspective offered by Thompson and Knaus [1] and the article by Myoshi et al. [10] touch on the need for clinical and mechanistic studies of interventions into neurogenetic disorders. With respect to Mabry syndrome, Thompson and Knause review the data supporting the use of pyridoxine to treat seizures [12] in some, but not all, patients with HPMRS2, HPMRS3, HPMRS4, and HPMRS5 forms of the disorder [13,14,15,16]. This is discussed in the context of studies suggesting that animal models of GPIBDs [17,18] may facilitate mechanistic investigations of this and other medical interventions. Although limited, the evidence of putative lysosomal storage in Mabry syndrome phenotypes, including HPMRS2 and 3 [19,20,21,22,23], may suggest pre-clinical endpoints if identified in model animals. By contrast, the mechanistic study by Myoshi et al. [10] of the novel general anesthetic remimazolam that has an unknown safety profile in malignant hyperthermia (MH) serves as a reminder that therapeutic studies for DDs lag those of other neurogenetic disorders. Myoshi et al. [10] used myotubes from the skeletal muscle of patients with MH to examine the Ca^2+-^induced Ca^2+^ release (CICR) rate test in response to remimazolam, a ryanodine receptor 1 (RYR1) agonist. The authors reported that the EC_50_ for remimazolam was lowest for CICR-positive, *RYR1*-mutant patients. They suggest that the intracellular calcium in myotubes from MH patients was elevated by remimazolam at concentrations exceeding those used clinically and conclude that remimazolam may be safe in MH [10]. Greater mechanistic studies of neurogenetic and rare genetic DD treatments are clearly warranted [1].

Finally, Morganthau et al. [11] report on the RARE Compassion Program, the first international educational program to pair medical students with rare disease patients in order to enhance the rare disease training of clinicians. The authors retrospectively reviewed responses from 334 student participants registered between 2014 and 2018. The findings of the RARE Compassion Program have implications for clinician training in that greater exposure of students to this group of patients may improve their understanding of important and often neglected DD patient perspectives. It is important to identify strategies that promote clinical engagement of genetic counseling and medical professionals that will allow the best delivery of services that are now possible as a result of the kind of advances presented in this Special Issue.

## Figures and Tables

**Figure 1:**
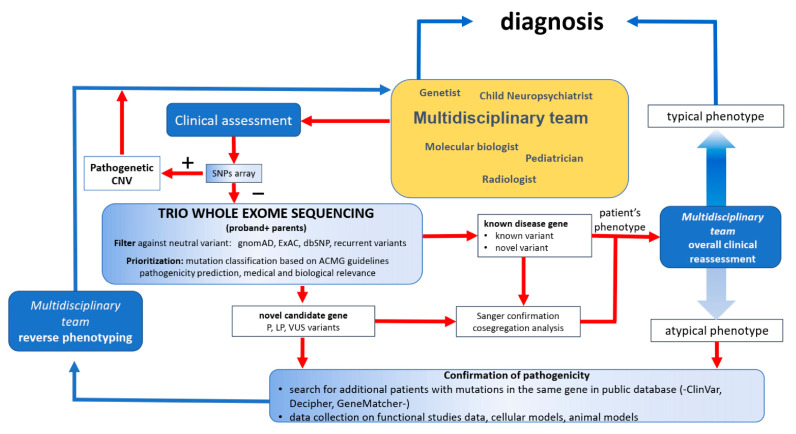
Schematic of the diagnostic procedure for developmental disabilities. Adapted from Musante et al. [2]. Abbreviations: SNP: single nucleotide polymorphism; CNV: copy number variation; gnomAD: the Genome Aggregation Database; ExAC: Exome Aggregation Consortium; dbSNP: the Single Nucleotide Polymorphism Database; ACMG: the American College of Medical Genetics and Genomics; P: pathogenic; LP: likely pathogenic; VUS: variant of unknown significance.
